# Six-minute walk test in children and adolescents with renal diseases: tolerance, reproducibility and comparison with healthy subjects

**DOI:** 10.6061/clinics/2016(01)05

**Published:** 2016-01

**Authors:** Flávia Tieme Watanabe, Vera Herminia Kalika Koch, Regina Celia Turola Passos Juliani, Maristela Trevisan Cunha

**Affiliations:** Instituto da Criança do Hospital das Clínicas da Faculdade de Medicina da Universidade de São Paulo,; I Divisão de Fisioterapia, IIUnidade de Nefrologia Pediátrica, São Paulo/, SP, Brazil

**Keywords:** Exercise Testing, Exercise Tolerance, Chronic Renal Failure, Renal Dialysis, Kidney Transplantation

## Abstract

**OBJECTIVES:**

To evaluate exercise tolerance and the reproducibility of the six-minute walk test in Brazilian children and adolescents with chronic kidney disease and to compare their functional exercise capacities with reference values for healthy children.

**METHODS:**

This cross-sectional study assessed the use of the six-minute walk test in children and adolescents aged 6-16 with stage V chronic kidney disease. For statistical analysis of exercise tolerance, including examinations of correlations and comparisons with reference values, the longest walked distances were considered. The reproducibility of the six-minute walk test was assessed using intraclass correlation coefficients.

**RESULTS:**

A total of 38 patients (14 females and 24 males) were evaluated, including 5 on peritoneal dialysis, 12 on hemodialysis and 21 who had undergone renal transplantation, with a median age of 11.2 years (6.5-16). The median walked distance was 538.5 meters (413-685) and the six-minute walk test was found to be reproducible. The walked distance was significantly correlated with age (r=0.66), weight (r=0.76), height (r=0.82), the height Z score (r=0.41), hemoglobin (r=0.46), hematocrit (r=0.47) and post-test systolic blood pressure (r=0.39). The chronic kidney disease patients predicted walked distance was 84.1% of the reference value according to age, 90.6% according to age-corrected height and 87.4% according to a predictive equation.

**CONCLUSIONS:**

The stage V chronic kidney disease patients had a significantly decreased functional exercise capacity, as measured by the six-minute walk test, compared with the healthy pediatric reference values. In addition, the six-minute walk test was shown to be well tolerated, reliable and applicable as a low-cost tool to monitor functional exercise capacity in patients with renal disease.

## INTRODUCTION

The six-minute walk test (6MWT) is a submaximal test that assesses the functional exercise capacities of healthy subjects or those with a chronic disease, as well as the effects of interventions, such as pulmonary rehabilitation or drug therapies. It can predict prognosis, mortality and morbidity in adults and children with lung and/or heart diseases 1-3.

The 6MWT has been used to assess exercise tolerance in children with chronic lung diseases, such as cystic fibrosis 4,5 and pulmonary hypertension 6 and in those on hemodialysis 7. Geiger et al. 8 evaluated the 6MWT in 528 healthy Caucasian children and adolescents aged 3-18 years, establishing reference values and formulating predictive equations for this population.

A study with of children with stage V chronic kidney disease (CKD) has used the 6MWT to demonstrate decreased exercise tolerance 9,10, which appears to be caused by several factors, such as anemia, metabolic acidosis, electrolyte imbalance, osteopenia, growth deficiency, malnutrition, inactivity, uremic muscle dysfunction and associated comorbidities 11-13.

Takken et al. 12 compared the 6MWT walked distances in Caucasian children and adolescents (8-18 years old) on peritoneal dialysis with the reference values. These patients exhibited a reduced exercise tolerance. The findings of this study suggest that the 6MWT could be used as a clinical tool to monitor functional exercise capacity in children with kidney disease. A reduction in functional exercise capacity, as determined using the 6MWT, has also been demonstrated in a Brazilian study of children and adolescents aged 6-16 with non-dialytic CKD 14.

Kohl et al. 15 evaluated 52 adult patients on hemodialysis for 12 years to determine the prognostic value of the 6MWT. They found that the 6MWT walked distance was a mortality predictor and verified an increase in life expectancy of approximately 5.3% for every 100 additional meters of the walked distance.

Guillén et al. 4, Cunha et al. 5 and Gulmans et al. 16 have demonstrated the reproducibility of the 6MWT in cystic fibrosis patients. However, its reproducibility has not been evaluated in pediatric CKD patients. The aims of this study were to evaluate exercise tolerance and the reproducibility of the 6MWT in Brazilian children and adolescents with stage V CKD and to compare the functional exercise capacities of these patients with internationally accepted reference values for healthy children.

## METHODS

This cross-sectional study was approved by the human ethics committee of Hospital das Clínicas da Faculdade de Medicina da Universidade de São Paulo, Brazil. Written informed consent was obtained from all subjects or their parents.

The sample size was calculated considering an alpha error of 0.5, a sensitivity of 0.8 and a confidence interval of 95%, demonstrating that the inclusion of at least 10 participants was necessary, according to Rollin Brant 17.

Clinically stable children and adolescents of both genders who were between the ages of 6-16, on peritoneal dialysis, on hemodialysis or at least three months post-kidney transplantation were included. Children with neurological, orthopedic, cardiac or respiratory disease were excluded, as well as those with unstable angina and those who had suffered myocardial infarction during the previous month, according to the contraindications of the American Thoracic Society (ATS) 18. The evaluation of 38 children and adolescents with stage V CKD using the 6MWT was performed between July 2011 and November 2012.

All of the children and adolescents underwent anthropometric measurements (weight and height) before participating in the 6MWT. Height and body mass index (BMI) Z scores were calculated using WHO AnthroPlus software 19. Systolic and diastolic blood pressure (SBP/DBP) Z scores were calculated according to the National Institutes of Health (NIH) guidelines 20. The 6 MWT was performed twice according to the ATS guidelines 18, with a rest interval of 30 minutes between tests, on a leveled 30 meter-long indoor corridor with markings at 1 meter intervals. The walked distance was measured in meters and the test with the longest distance was selected. During the test, the patients received standardized verbal encouragement to walk as far as possible and they were allowed to rest if necessary, but the chronometer was not stopped. The following measurements were obtained at rest and at the end of the test: heart rate (HR; POLAR^®^), respiratory rate (RR; STOP WATCH^®^ chronometer), pulse oxygen saturation (SpO_2_; pulse oximeter NONIN^®^), blood pressure (BP; BIC^®^ aneroid sphygmomanometer) and the perception of dyspnea, which was measured using the Modified Borg Scale 21,22, with scores ranging from 0 (no perception of dyspnea) to 10 points (maximum perception of dyspnea).

The laboratory results for hemoglobin, hematocrit, urea, creatinine, total calcium, ionic calcium, phosphate and parathyroid hormone concentrations (within one month before and after the 6MWT date) were obtained from the medical records, as well as the dates of transplantation, hemodialysis and peritoneal dialysis sessions.

The patients' walked distances were compared with age–gender reference values and with predicted walked distances using the prediction equations of Geiger et al. 8. This comparison was performed in the following three modes: with pairing by chronological age (individuals of the same age), with pairing by height with correction for age at the 50^th^ percentile of the World Health Organization (WHO) growth curve 23 and with pairing using the walked distance predictive equation (including sex, age and height). Statistical analysis was performed using the percentages of the predicted walked distances for comparisons.

SPSS software for Windows (SPSS Inc. Release 14 standard version) was used for statistical analysis, with a significance level of *p*<0.05. The test with the longest walked distance was considered for statistical analysis and the median and range were calculated. Spearman's test was used to study the correlation between the longest walked distance and age, anthropometric measurements, hemoglobin, hematocrit and post-test SBP. The sample characteristics did not allow for multiple linear regression analysis.

The Mann-Whitney test was used to compare the quantitative variables between the patient groups (transplanted and dialysis). The intraclass correlation coefficient (ICC), standard error of measurement and smallest detectable change assess the reproducibility of the 6MWT. The Wilcoxon test was used to compare the 6MWT measurements (at rest and at the end of the test) and the walked distances of the study group with the age-gender reference values. The results of this comparison are presented as absolute values and percentages of the predicted values.

## RESULTS

This study included 38 children and adolescents (24 males and 14 females) with a median age of 11.2 (6.5-16) who were on peritoneal dialysis (N=5) or hemodialysis (N=12) or had undergone renal transplantation (N=21). The patients' median time on dialysis and time since renal transplantation were 2 years (1-8 years) and 2 years (5 months-4 years), respectively. Table 1 shows the anthropometric and clinical data of the studied population.

In this study, 50% of the population (N=19) had height measurements that were below the third percentile for age (WHO) 23. The post-transplant patients were significantly taller than the dialytic patients (hemodialysis and peritoneal dialysis; *p*=0.041). There were no adverse events associated with the 6MWT and no patient needed to rest during the test.

The median longest walked distance was 538.5 m (413-685 m) (Table 2) for the total group. Patient subgroup analysis showed that the post-transplant participants walked a longer distance than the dialysis patients; however, no significant differences were detected in the other 6MWT measurements (586 m (442-685 m) x 500 m (413-598 m), (*p*=0.002)).

In the present study, 12 (32%) patients, including 4 post-transplant and 8 dialysis patients aged 6-14, demonstrated difficulty in understanding the Modified Borg Scale for the perception of dyspnea and were excluded from the statistical analysis of this item.

**Figure 1:**
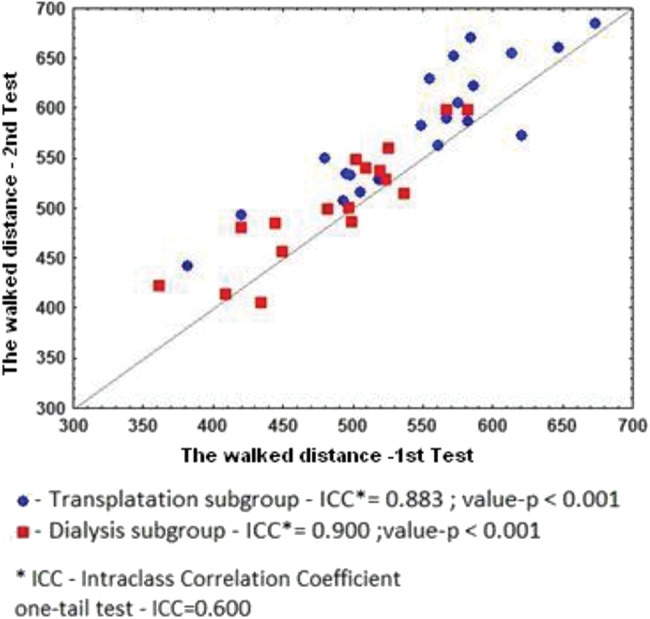
Reproducibility of the walked distance between the first and second 6-minute walk tests.

The walked distance on the second 6MWT was generally longer than that of the first test (538.5 m (405-685 m) x 519.5 m (362-674 m); *p*<0.001), with the exception of 4 patients who performed better on the first test. The 6MWT reproducibility was confirmed for all variables (ICC>0.4), except for the post-test SpO_2_ (ICC=0.15) and pre-test dyspnea (ICC=0.13) (Table 3). The standard error of measurement for the total group was 21.8 m and the smallest detectable change was 60.5 m. Figure 1 demonstrates the reproducibility between the first and second 6MWTs for each variable.

Our data revealed positive correlations between the longest walked distance and age (r=0.66, *p*<0.001), weight (r=0.76, *p*<0.001), height (r=0.82, *p*<0.001), the Z score for height (r=0.41, *p*=0.010), hemoglobin (r=0.46, *p*=0.003), hematocrit (r=0.47, *p*=0.003) and post-test SBP (r=0.39, *p*=0.015).

Table 4 shows the walked distances of our study subjects and those of the subjects in the Geiger et al. study 8 according to sex and age.

As shown in Table 5, the stage V CKD patients walked shorter distances compared with the healthy population reference values of Geiger et al. 8 (*p*<0.001).

## DISCUSSION

In the present study, children and adolescents with stage V CKD performed the 6MWT twice without adverse events, showing that this test is a simple, quick, inexpensive, reliable and safe method to assess functional exercise capacity in this population, as has already been suggested in the literature 9-14. Other alternatives for the evaluation of functional exercise capacity are maximal exercise tests performed on a bicycle or treadmill; however, such methodologies require expensive equipment.

In the present study, the youngest patients had no difficulty in understanding or in performing the 6MWT. Our findings are in agreement with those of Lammers et al. 3, who assessed the 6MWT in 4- and 5-year-old children and concluded that with clear direction and active encouragement as standardized by the ATS 18, this test can be applied in this age group, with the same walked distance variability as in older age groups.

In our study, the walked distance of the second 6MWT was longer than that of the first test, as described in the ATS consensus 18, reinforcing the need to perform two tests.

The 6MWT walked distances in the present study were significantly shorter than the reference values for healthy children published by Geiger et al. 8. Our dialysis patient subgroup showed a similar walked distance on the 6MWT (81.6% of the walked distance predicted value) compared to that described by Takken et al. 12 (83% of the predicted value). Although the BMI Z scores of our dialysis subgroup were normal, other factors could have contributed to the reduction in functional exercise capacity, such as decreased height, anemia, a reduced renal glomerular filtration rate and a high parathyroid hormone level.

Cury et al. 24 have demonstrated significant differences in the 6MWT walked distance among adults in a control group, hemodialysis group, and renal transplant group, suggesting that renal transplantation improves this distance without promoting full recovery of the functional exercise capacity. These results corroborate those of the present study, which demonstrated that renal transplantation patients walked a longer distance compared to dialysis patients; however, their median walked distance was still 10.5% lower than the age-gender reference value (*p*<0.001). In addition to the factors that may have contributed to the low functional exercise capacities of the CKD patients, the use of steroid therapy for kidney transplantation immunosuppression may also have had a negative impact on muscle fibers, resulting in a decrease in muscle protein synthesis and impairing oxidative metabolism 24.

Studies have shown that physical inactivity is prevalent in the pediatric CKD population. This inactivity occurs due to the need to avoid contact sports, a lower exercise tolerance, anemia, vascular catheter- and arteriovenous fistula-related restriction of physical activity and time spent on hemodialysis 9-14. Most of the children in the present study did not routinely exercise, and this may have contributed to their poor functional exercise capacities, although physical activity levels were not assessed in this study.

After a median walked distance of 538.5 m on the 6MWT, the study participants exhibited increases in the HR, BP, RR and perception of dyspnea measurements compared to the baseline values, which is expected as a normal physiological response related to physical exertion. These findings corroborate with those of Coelho et al. 14, who demonstrated that CKD patients on conservative treatment covered a shorter walked distance (560 m) compared with healthy control children (724 m) and had a higher BP, RR and decreased perception of dyspnea at the end of the test. A partial limitation of our study was the lack of a control group, although age-gender reference values for healthy children were used.

The 6MWT also depends on the physical effort of patients to walk longer distances. The present study demonstrated a correlation between the walked distance and post-test SBP and this finding may reflect this physical effort or the presence of a condition causing poor BP control.

According to the ATS 18 and previous studies 8,25, a correlation exists between height and the walked distance on the 6MWT. Multiple factors are responsible for growth retardation in children with CKD, including persistent metabolic acidosis, CKD-associated metabolic bone disease, vitamin D deficiency, protein-calorie malnutrition and hormonal alterations in the GH-IGF1 axis 26. All of these effects may result in a lower height and shorter walked distance on the 6MWT. In line with these studies, we demonstrated correlations between the walked distance and height, age and weight. A greater height usually results in increased lower limb length, a bigger step and greater speed. Oliveira et al. 25 have suggested that variation in the walked distance on the 6MWT can be predicted more accurately with an equation including the lower limb length.

Although there are no data on the positive or negative effects of interventions on the 6MWT walked distances of CKD pediatric patients, the present study has demonstrated that the smallest detectable change is 60.5 m. This suggests that a measurement of above 60.5 m in the walked distance for this population can be considered a significant outcome.

The functional exercise capacity depends on a perfect interaction between the respiratory and cardiovascular systems and peripheral muscles. The hematocrit directly influences the active transport of oxygen and carbon dioxide in peripheral muscles and can affect the walked distance. A study of adults on hemodialysis (the Canadian Erythropoietin Study Group) 27 has demonstrated that the use of erythropoietin significantly increases the hemoglobin and hematocrit levels but does not affect the 6MWT walked distance. However, in our study, a correlation was found between the walked distance and hemoglobin, as well as between the walked distance and the hematocrit level, in agreement with Takken et al. 12. The hemoglobin and hematocrit levels may be important predictors of the 6MWT walked distance for stage V CKD patients.

No differences were detected between the walked distances on the two successive tests conducted under the same clinical conditions (Table 3, Figure 1), indicating that the 6MWT is reproducible. These results corroborate with those of Guillen et al. 4, Cunha et al. 5 and Gulmans et al. 16, who evaluated children with cystic fibrosis and found that they were able to perform this test properly and repeatedly. All of the 6MWT measurements were reproducible in this study, except for the post-test SpO_2_ and pre-test perception of dyspnea. Although significant differences were observed between the two post-test SpO_2_ values, they are clinically comparable. Despite the validation of the Modified Borg Scale for perception of dyspnea for use in children, Morinder et al. 28 have reported that obese children have difficulty in understanding its use. In our study, 12 patients aged 6-14 were not able to understand this scale. We believe that this difficulty in understanding the Modified Borg Scale may explain the lack of reproducibility of this measurement.

Our patients' walked distances were compared with reference values for healthy children and adolescents according to chronological age, according to age-corrected height and using the walked distance predictive equation. These three comparisons demonstrated that the stage V CKD patients walked a shorter distance. A higher percentage difference in the walked distance was observed in the comparison according to chronological age, reflecting the substantial growth retardation characteristic of CKD pediatric patients. Significant but lower percentage differences in this distance were detected in the comparisons according to age-corrected height and using the walked distance predictive equation of Geiger et al. 8, which takes into account gender, height and age. These results suggest that growth deficit is not the only performance-limiting factor that decreases the walked distance of stage V CKD pediatric patients on the 6MWT. The three modes used in the present study to compare the patients' walked distances with reference values from healthy children and adolescents can be useful in the monitoring of the clinical and functional statuses of stage V CKD patients. However, we suggest that the use of the predictive equation may be more advantageous because it incorporates two important variables: age and height.

In addition, the 6MWT provides important measurements for assessments of the global and integrated responses of the systems involved during exercise, especially HR and BP. Most activities of daily living are performed at a submaximal level of exertion. Thus, the submaximal test may be an important tool for assessing the clinical and functional statuses of stage V CKD patients. Our finding of the lower functional exercise capacities of children and adolescents with stage V CKD is supported by other studies in the literature; however, the 6MWT is not routinely performed in this patient population. We suggest that this test should be repeated at least once a year.

We also suggest that a training program could be used for children with stage V CKD. This training program should be carried out and monitored by a qualified multidisciplinary team to ensure for adequate monitoring of HR and BP, implementation of arteriovenous fistula- and catheter-related restrictions and avoidance of contact activities.

Further studies of children and adolescents with stage V CKD should be performed to help determine the best type of exercise, the ideal exercise intensity and the achievable results from exercise training.

Children and adolescents with stage V CKD have significantly poorer functional exercise capacities, as measured by the 6MWT, compared with the pediatric reference values. This test is well tolerated and reliable, which favors its application as a low-cost tool to monitor functional exercise capacity in patients with renal disease.

## AUTHORS CONTRIBUTION

Watanabe FT and Cunha MT conceived and designed the study and were responsible for the data acquisition, analysis and interpretation, manuscript drafting and critical revision. Koch VH, Juliani RC were responsible for the study design and critical revision.

## Figures and Tables

**Table 1:** Anthropometric and clinical data for children and adolescents with stage V chronic kidney disease.

	Total group (N=38)	Post-transplant group (N=21)	Dialysis group (N=17)	*p*
	Median (min-max)				
**Age (years)**	11.2 (6.5-16.0)	11.4 (6.5-16.0)	10.3 (6,6-16.0)	0.913
**Weight (kg)**	28.6 (17.2-63.7)	33.2 (17.2-63.7)	26.0 (17.9-46.3)	0.014*
**Height (m)**	1.3 (0.9-1.6)	1.4 (0.9-1.6)	1.3 (1.1-1.5)	0.041*
**Height (Z score)**	-1.9 (-5.7-1.8)	-1.0 (-5.7-1.8)	-2.7 (-5.5-0.7)	0.019*
**BMI (kg/m^2^)**	17.5 (13.4-27.6)	18.6 (15.3-27.6)	16.2 (13.4-24.8)	0.007*
**BMI (Z score)**	-0.1 (-2.3-3.1)	0.4 (-1.5-3.1)	-0.5 (-2.3-2.2)	0.034*
**Hemoglobin (g/dL)**	11.5 (7.6-15.8)	12.4 (10.0-15.8)	10.4 (7.6-13.3)	0.004*
**Hematocrit (%)**	34.5 (23.0-40.0)	37.0 (31.0-40.0)	33.0 (23.0-39.0)	0.029*
**Urea (mg/dL)**	38.0 (14.0-192.0)	35.0 (21.0-82.0)	55.0 (14.0-192.0)	0.145
**Creatinine (mg/dL)**	1.2 (0.4-10.6)	0.8 (0.4-1.9)	3.7 (1.7-10.6)	<0.001*
**Calcium (mg/dL)**	9.5 (8.0-10.1)	9.5 (8.6-10.1)	9.4 (8.0-10.1)	0.263
**Ionic calcium (mmol/L)**	1.2 (1.0-1.4)	1.2 (1.1-1.3)	1.2 (1.0-1.4)	0.515
**Phosphate (mg/dL)**	4.8 (2.5-6.2)	4.9 (3.6-5.9)	4.4 (2.5-6.2)	0.167
**Parathyroid hormone (pg/mL)**	83.5 (3.0-1,150.0)	82.0 (3.0-211.0)	110.0 (3.0-1,150.0)	0.348
**SBP (mmHg)**	110 (90-140)	120 (100-130)	110 (90-140)	0.378
**SBP (Z score)**	1.3 (-1.1-3.6)	1.2 (-0.6-2.6)	1.3 (-1.1-3.6)	0.953
**DBP (mmHg)**	80 (60-90)	80 (60-90)	80 (60-90)	0.867
**DBP (Z score)**	1.5 (-0.5-3.0)	1.4 (-0.1-2.9)	1.7 (-0.5-3.0)	0.902

Values are expressed as the median and minimum-maximum values. BMI: body mass index; SBP: systolic blood pressure; DBP: diastolic blood pressure

**<?ENTCHAR ast?>:** Statistically significant differences between subgroups (*p*<0.05) (Mann-Whitney test).

**Table 2:** The longest walked distance on the 6-minute walk test for stage V chronic kidney disease patients.

	Total group (N=38)		Post-transplant (N=21)		Dialysis (N=17)	
	Pre	Post	Pre	Post	Pre	Post
**HR (bpm)**	94 (70-133)	147 (111-187)*	92 (70-133)	147 (127-183)*	94 (77-130)	149 (111-187)*
**SpO_2_ (%)**	98 (95-100)	97 (94-100)*	98 (95-99)	97 (94-98)**	98 (97-100)	97 (96-100)**
**SBP (mmHg)**	110 (90-140)	130 (110-180)*	120 (100-130)	140 (120-180)*	110 (90-140)	130 (110-160)*
**DBP (mmHg)**	80 (60-90)	80 (60-110)*	80 (60-90)	80 (60-110)**	80 (60-90)	80 (60-110)**
**MBP (mmHg)**	90 (70-100)	100 (80-120)*	90 (70-100)	100 (90-120)*	90 (70-100)	100 (80-120)*
**RR (bpm)**	20 (12-28)	32 (24-44)*	20 (12-25)	32 (24-40)*	20 (16-28)	32 (24-44)*
**Perception of dyspnea**	0 (0-1)	2 (0-10)*	0 (0-1)	0.5 (0-10)**	0 (0-0.5)	3 (0-5)**
**Walked distance (m)**	-	538.5 (413-685)	-	586 (442-685)	-	500 (413-598)†

Values are expressed as the median and minimum-maximum values; HR: heart rate in beats per minute (bpm); SpO_2_: pulse oxygen saturation (%); SBP: systolic blood pressure in mmHg; DBP: diastolic blood pressure in mmHg; MBP: mean diastolic blood pressure in mmHg; RR: respiratory rate in breaths per minute (bpm); and perception of dyspnea, determined using the Modified Borg Scale.

**<?ENTCHAR ast?>:** Significant difference between pre- and post-6MWT (*p<*0.001) (Wilcoxon test);

**<?ENTCHAR ast?><?ENTCHAR ast?>:** **significant difference between pre- and post-6MWT (*p*<0.05) (Wilcoxon test);

**<?ENTCHAR dagger?>:** †significant difference between subgroups in walked distance (*p*<0.05) (Mann-Whitney test).

**Table 3:** Reproducibility of the 6-minute walk test in stage V chronic kidney disease patients.

	Total group (N=38)			Post-transplant (N=21)			Dialysis (N=17)		
	6MWT 1	6MWT 2	ICC	6MWT 1	6MWT 2	ICC	6MWT 1	6MWT 2	ICC
**HR pre (bpm)**	94.5 (69-135)	93.5 (70-133)	0.90	93 (69-128)	92 (70-133)	0.86	95 (77-135)	94 (83-130)	0.94
**HR post (bpm)**	150 (111-187)	147 (110-187)	0.63	148 (122-172)	147 (127-183)	0.64	154 (111-187)	149 (110-187)	0.63
**SpO_2_**pre** (%)**	98 (95-100)	98 (95-100)	0.56	98 (96-99)	98 (95-99)	0.67	98 (95-100)	98 (96-100)	0.47
**SpO_2_**post** (%)**	97 (91-100)	97 (94-100)	0.15	97 (94-99)	97 (94-98)	0.32	97 (91-100)	97 (96-100)	0.08
**SBP pre (mmHg)**	110 (90-140)	110 (90-130)	0.75	120 (100-130)	120 (100-130)	0.70	110 (90-140)	110 (90-130)	0.78
**SBP post (mmHg)**	140 (110-180)	130 (110-180)	0.81	140 (120-180)	140 (120-180)	0.77	130 (110-160)	130 (110-160)	0.81
**DBP pre (mmHg)**	75 (60-90)	80 (60-90)	0.69	70 (60-90)	80 (60-90)	0.77	80 (60-90)	80 (60-90)	0.60
**DBP post (mmHg)**	80 (60-120)	80 (60-110)	0.63	80 (60-100)	80 (60-110)	0.65	80 (60-120)	80 (60-110)	0.80
**RR pre (rpm)**	20 (16-28)	20 (12-28)	0.51	20 (16-24)	20 (12-25)	0.25	20 (16-28)	20 (16-28)	0.77
**RR post (rpm)**	32 (20-44)	32 (24-44)	0.67	32 (24-44)	32 (24-40)	0.50	32 (20-44)	32 (24-44)	0.84
**Dyspnea pre**	0 (0-0.5)	0 (0-1)	0.13	0 (0-0.5)	0 (0-1)	0.21	0 (0-0.5)	0 (0-0.5)	0.13
**Dyspnea post**	3.5 (0-10)	2 (0-10)	0.57	3 (0-10)	0.5 (0-10)	0.52	4 (0-9)	3.5 (0-7)	0.73
**Walked distance (m)**	519 (362-674)	538.5 (405-685)	0.91	561 (382-674)	583 (442-685)	0.89	499 (362-583)	500 (405-598)	0.90

Pre- and post-6MWT values are expressed as the median and minimum-maximum values, ICC (intraclass correlation coefficient). HR: heart rate in beats per minute (bpm); SpO_2_: oxygen pulse saturation (%); SBP: systolic blood pressure in mmHg; DBP: diastolic blood pressure in mmHg; RR: respiratory rate in breaths per minute (bpm); perception of dyspnea, determined using the Modified Borg Scale; and walked distance in meters (m).

**Table 4:** Distribution of stage V chronic kidney disease patients' walked distances compared with age–gender reference values (Geiger et al. (8)).

Sex	Age category (years)	n	CKD walked distance (m)	Walked distance reference value (m)
**Male**	6 to 8	6	496	584.0
		9 to 11	5	537	667.3
		12 to 15	10	625	701.1
		16	3	598	727.6
**Female**	6 to 8	4	509	578.3
		9 to 11	8	504	655.8
		12 to 15	2	549	657.6
		16	0	-	660.9

Values are expressed as the median.

**Table 5:** Comparison of stage V chronic kidney disease patients' median 6-minute walk test walked distances with age-gender normative values for healthy population (Geiger et al. (8)).

		Walked distance in meters and percentage of predicted value		
	Walked distance (m)	Chronological age	Age-corrected height	Predictive equation
**Total group**	538.5 (413-685)	640.3 (84.1%)*	594.4 (90.6%)*	616.1 (87.4%)*
**Post-transplant subgroup**	586.0 (442-685)	653.3 (89.7%)*	629.4 (93.1%)*	636.3 (92.1%)*
**Dialysis subgroup**	500.0 (413-598)	639.4 (78.2%)*	596.7 (83.8%)*	612.7 (81.6%)*

Walked distance values are expressed as the median (minimum-maximum), and predicted walked distances are expressed as the median (percentage of predicted value (%)).

**<?ENTCHAR ast?>:** *Significant difference between stage V CKD patient walked distance and reference value (*p*<0.001) (Wilcoxon test).
